# Metabolic reprogramming through mitochondrial biogenesis drives adenosine anti-inflammatory effects: new mechanism controlling gingival fibroblast hyper-inflammatory state

**DOI:** 10.3389/fimmu.2023.1148216

**Published:** 2023-06-07

**Authors:** Nathalie Paladines, Shantiece Dawson, Weston Ryan, Rogelio Serrano-Lopez, Regina Messer, Yuqing Huo, Christopher W. Cutler, Erivan S. Ramos-Junior, Ana Carolina Morandini

**Affiliations:** ^1^ Department of Oral Biology and Diagnostic Sciences, Dental College of Georgia, Augusta University, Augusta, GA, United States; ^2^ Department of Cellular Biology and Anatomy, Medical College of Georgia, Augusta University, Augusta, GA, United States; ^3^ Vascular Biology Center, Augusta University, Augusta, GA, United States; ^4^ Department of Periodontics, Dental College of Georgia, Augusta University, Augusta, GA, United States

**Keywords:** fibroblasts, adenosine, mitochondrial biogenesis, inflammation, metabolism

## Abstract

**Introduction:**

Fibroblasts are the dominant stromal cells in the gingival lamina propria with a well-established relevance in regulation of inflammation, and in innate immunity. This is exemplified by their hypersecretion of CXCL8, enhancing leukocyte infiltration in chronic and sustained inflammatory conditions. We have previously shown adenosine to be a key metabolic nucleoside that regulates stromal inflammation, but the underlying mechanisms linking adenosine to the metabolic status of fibroblasts and to the resultant inflammatory response are unclear. This study examined, by seahorse real-time cell metabolic analysis, the bioenergetics of the stromal fibroblast response to extracellular adenosine and IL-1β, focusing on CXCL8 secretion by primary human gingival fibroblasts (HGF).

**Methods:**

Markers of the glycolytic pathway and mitochondrial biogenesis were tracked through immunoblot. Further, the influence of adenosine on mitochondrial accumulation was measured by uptake of MitoTracker Red fluorescent probe and assessment of the role of FCCP (a mitochondrial uncoupler) in CXCL8 secretion and mitochondrial accumulation.

**Results:**

Our results show that the anti-inflammatory response of HGF to extracellular adenosine, typified by reduced CXCL8 secretion, is mediated by mitochondrial oxidative phosphorylation, reflected in higher oxygen consumption rate (OCR). In the presence of IL-1β, adenosine-treated cells induced higher ATP production, basal respiration and proton leak compared to IL-1β without adenosine. Surprisingly, adenosine had no additional effect on the IL-1β-induced higher glycolysis rate demonstrated by the extracellular acidification rate (ECAR). In addition, the higher OCR in adenosine-stimulated cells was not due to the mitochondrial fuel dependency or capacity, but due to an increase in mitochondrial biogenesis and accumulation in the cells with concomitant decrease in mitophagy-required p-PINK1 marker. We detected the accumulation of functional mitochondria with increased activation of the AMPK/SIRT1/PGC-1α pathway. The adenosine-induced uptake of MitoTracker was abrogated by PGC-1α inhibition with SR-12898. In addition, the adenosine effects on reduced CXCL8 were ablated by treatment with FCCP, a potent uncoupler of mitochondrial oxidative phosphorylation.

**Conclusion:**

Our findings reveal a key role for mitochondrial bioenergetics in regulation of CXCL8-mediated inflammation by HGF through the adenosine/AMPK/SIRT1/PGC-1α axis. Therapeutically targeting this pathway in gingival fibroblasts might be a promising future strategy to modulate stromal-mediated sustained hyper-inflammatory responses.

## Introduction

Human gingival fibroblasts (HGF), are the most abundant cells in the gingival lamina propria, and play a vital role in the development and progression of periodontitis through their response to periodontal pathogens (1) and inflammatory cytokines (2). These factors are abundant in the inflammatory milieu of untreated disease. Among the inflammatory markers relevant to periodontal immunobiology, interleukin (IL)-1β is most prominent, as it is a strong stimulator of bone resorption (3), as reviewed previously (4). IL-β is a key inflammatory mediator in the pathogenesis of periodontitis (5–8), and is one of the dominant cytokines detected in the gingival crevicular fluid of diseased patients (9); moreover, the IL-1 genotype has been implicated in susceptibility to severe disease manifestation (10). Previous study by our group showed that autocrine IL-1 signaling is necessary for leukocyte recruitment and keystone pathogen clearance (11). In this regard, our group and others have also demonstrated the importance of adenine nucleotides such as adenosine triphosphate (ATP) and its by-product adenosine, as endogenous regulators of inflammation and essential mediators for the balance between cell damage and repair (12, 13).

Extracellular ATP (eATP) acts as a mediator of stress signaling to cells during times of infection or inflammation (14). Adenosine is a purine nucleoside which limits mucosal inflammation (15) being well known for its immunosuppressive functions. eATP is not very stable and can be easily hydrolyzed by the action of ectonucleotidases, particularly under pathological conditions. This increases the adenosine concentration (16). Our previous research demonstrated the degradation of ATP to adenosine through ectonucleotidase CD73 reduced chemokine CXCL8 secretion induced by IL-1β in HGF, effectively decreasing inflammation in these cells through adenosine receptors (17). Adenosine receptors are classified into four subtypes, A1, A2A, A2B, and A3, all activated by extracellular adenosine, playing central roles in a broad range of physiological processes, including modulation of the immune system (18). A2AR is one of the most well characterized G-protein coupled receptors and A2A and A2B are broadly recognized as critical to the immune functions of adenosine (19). Physiologically, the concentration of extracellular adenosine in healthy tissues is low (20), but it can rapidly escalate from nanomolar to micromolar concentrations depending on cellular stress, such as injury, hypoxia or inflammation (21, 22). Therefore, a rise in local adenosine levels contributes to tissue protection against damage (23) from hyper-inflammatory response or excessive immune activation (24).

An increase in phosphorylated adenosine monophosphate-activated protein kinase (pAMPK) was observed after IL-1β stimulation and adenosine receptor activation, suggesting the anti-inflammatory effects of adenosine exposure are dependent on the upregulation of pAMPK (17). AMPK is an energy sensor in the body and thus acts as a major regulator of cell energy metabolism. AMPK can sense changes in the ATP-to-AMP ratio and is activated when intracellular levels of ATP decrease. Primarily, AMPK acts to inhibit anabolism and stimulate catabolism to restore intracellular ATP levels. Studies have shown AMPK is involved in the activation of genes related to mitochondrial biogenesis, which is a self‐renewal route by which new mitochondria are generated from existing stores. Among the specific molecules involved in this fine‐tuning, the peroxisome proliferator‐activated receptor‐γ coactivator (PGC)‐1α is the main regulator of mitochondrial biogenesis (25). In fact, AMPK and PGC-1α have a unique relationship in regulating mitochondrial biogenesis (25, 26) because of a strong overlap in the genes transcriptionally regulated by AMPK and those by PGC-1α. Additionally, there is evidence that Sirtuin 1 (SIRT1), a histone deacetylase works in concert with AMPK and PGC-1α to regulate cell metabolism (26). SIRT1 interacts with PGC-1α and promotes its transcriptional activity through deacetylation, but whether and how adenosine can eventually modulate all these sensors affecting cell energy metabolism and ultimately influencing inflammation is far from conclusive.

The participation of adenosine signaling in key metabolic pathways affecting the cellular energy outcome such as AMPK/SIRT1/PGC-1α pathway and consequently chemokine secretion is not clear, especially in gingival stromal cells. In fact, stromal cells have been showing an undeniable biological significance to promote or inhibit inflammation as protagonists in models of cancer microenvironment (27), dermal inflammation (28) and oral mucosa immunity (29). The exaggerated stromal cell responsiveness of fibroblasts is typified by hypersecretion of CXCL8 (29), which enhances leukocyte infiltration in periodontitis. Dysregulation of CXCL8 is a key feature of active periodontal inflammation (30, 31) and bone pathology (32). Understanding the pathways that regulate CXCL8 may provide vital clues for controlling inflammatory bone loss.

In this study, we examined the intersection of stromal inflammation, mitochondrial metabolism and adenosine signaling by examining anti-inflammatory adenosine effects on mitochondrial function and biogenesis and, conversely, mitochondrial feedback on inflammation, with an emphasis on metabolic markers that influence cellular homeostasis during the course of periodontal inflammation.

## Methods

### Cell culture and treatments

Primary human gingival fibroblasts (HGF-1) were previously established in the lab (IRB # 911778-10) and purchased from ATCC (CRL-2014). Cells were cultivated in Dulbecco’s Modified Eagle’s Medium (DMEM) supplemented with 10% fetal bovine serum and 100 UI/mL penicillin/streptomycin. HGF were cultured in a 5% CO_2_ incubator at 37°C. The growth media was replaced every 2-3 days. Experiments were performed using cells between the 4^th^ and 8^th^ passages, at approximately 80% confluence detailed in figure legends. The fibroblast phenotype was confirmed by positive staining for vimentin and FSP1. Viable cells were automatically counted using a cell counter and Trypan Blue staining and seeded in uniformity of cell distribution in OptiMEM medium the day before each experiment. For cell treatments, the following reagents were used: 1ng/mL human recombinant IL-1β (201LB005, R&D systems); 100μM Adenosine (A4036-5G, MilliporeSigma); 30μM EHNA (E114-25MG, MilliporeSigma), 1μM FCCP (S8276, Selleck Chemicals LLC) and 10μM SR-18292 (HY-101491, MedChem Express). Pre-treatment with drugs was performed 1 hour prior to the adenosine or EHNA+adenosine stimulation. EHNA was added 5min prior to adenosine. Adenosine was added 5 min prior to IL-1β and cells were incubated for the time points indicated in each figure legend.

### Antibodies

Western blotting experiments were performed using the following antibodies with respective dilutions: Antibodies from Abcam: A2AR (1:1000, ab151523); PFKFB3 (1:1000, ab181861); PGC-1alpha (1:1000, ab176328); mitobiogenesis cocktail [SDHA/MT-COX1] (1:250, ab123545). Antibodies from Cell Signaling: Phospho-AMPKα (Thr172) (40H9) (1:1000, #2535); AMPKα (D5A2) (1:1000, #5831); HRP Conjugate β-Tubulin (9F3) (1:1000, #5346); SIRT1 (D1D7) (1:1000, #9475); Hexokinase II (C64G5) (1:1000, #2867); PKM2 (D78A4) XP (1:1000, #4053); Phospho-PINK1 (Ser228) (1:1000, #46421).

### Metabolic analysis using seahorse technology

Metabolic characterization of HGF was performed using a Seahorse XFe96 Extracellular Flux Analyzer (Seahorse Bioscience). Briefly, 4 × 10^4^ cells were seeded into the Seahorse XF Cell Culture Microplate (Agilent Technology) in OPTiMEM, one day before the experiment. For analysis, cells were resuspended in XF assay media (Agilent Technology) supplemented with 10 mM glucose (Sigma-Aldrich), 1 mM pyruvate (Sigma-Aldrich), and 2 mM glutamine (Sigma-Aldrich). The Cell Mito Stress Test was performed using 1.5 μM oligomycin, 1.0 μM FCCP (carbonyl cyanide-p-trifluoromethoxy-phenyl-hydrazone), 0.5 μM rotenone, and 0.5 μM antimycin A (RotAA) purchased from Agilent Technologies. The Glycolysis Stress Test was performed using 1.0 μM oligomycin, 10mM Glucose, and 50 mM 2-Deoxy-D-glucose purchased from Agilent Technologies. All results were normalized per the total protein in each well after the assay using the BCA method (Pierce Protein Biology). Mitochondrial stress and glycolytic parameters were measured *via* Oxygen Consumption Rate (OCR) and Extracellular Acidification Rate (ECAR), respectively, in pmol/min/µg of protein. The Mito Fuel Flex Test was performed to measure the OCR and test dependency, capacity and flexibility of cells to oxidize glucose, glutamine or long-chain fatty acids as mitochondrial fuel. Metabolic parameters were exported and calculated according to the manufacturer’s instructions (Agilent Technologies) using the Seahorse Wave desktop software (Agilent Technologies).

### RNA isolation, cDNA synthesis and quantitative PCR

RNA was extracted from 1x10^5^ cells/well with the Invitrogen PureLink RNA Mini Kit (ThermoFisher Scientific) according to the manufacturer’s instruction. Briefly, samples were lysed in equal volumes of lysis buffer and stored in a -80°C freezer for a few hours. Then, samples were homogenized by vortexing and centrifuging for 5 minutes at 4°C at 12,000xg. After adding 70% ethanol, RNA was extracted and purified using a fast spin-column workflow and kit-provided wash buffers. All samples were eluted in 30 µl of RNase-Free water stored in a -80°C freezer. Reverse transcription was performed using a SuperScript IV VILO Master Mix (ThermoFisher Scientific) to obtain cDNA from Nanodrop read samples. The quantitative PCR was performed using the following inventoried Taqman assays: human CXCL1: Hs00236937_m1; human CXCL8: Hs00174103_m1 and human RPL13A: Hs03043885_g1 in a 10uL final volume using TaqMan Fast Advanced Master Mix in a StepOne Plus Real-Time PCR system (Applied Biosystems). Relative quantitation of the RPL13A reference gene versus the target gene was performed in duplex reactions and calculated using the comparative Ct (ΔΔCt) values to generate the RQ for each sample based on the established cycle threshold for each target. Analysis was performed using the StepOne Plus software and Graph Pad Prism.

### ELISA

The soluble secreted CXCL8 in the samples was measured by using a capture and a detection antibody using a Human IL-8/CXCL8 DuoSet ELISA (DY20805, R&D systems) and the respective reagents and plates provided by the DuoSet ELISA Ancillary Reagent Kit 2 (DY008B, R&D systems) according to the manufacturer’s instructions. Plates were read at the recommended wavelength in a Synergy H1 microplate reader (Biotek, Agilent Technologies).

### Western blotting

For WB analyses, cold RIPA buffer (ThermoFisher) was applied to extract total protein from 5x10^5^ cells/well in 6-well plates. The extracted proteins were measured using the BCA protein assay reagent kit (Pierce Protein Biology). An equal amount of total protein (10 μg of protein/lane) was then resolved with a 5–12% SDS-PAGE gel and electrotransferred onto a polyvinylidenedifluoride (PVDF) membrane (Bio-Rad) in a fast-transfer mode. The membranes were blocked with 5% non-fat dry milk in TBST (containing 0.05% Tween-20) and incubated overnight at 4°C with a primary antibody against each target (antibody dilution indicated in each figure legend). Following an incubation with an HRP-conjugated anti-rabbit or anti-mouse secondary antibody (1:25.000; abcam) at room temperature for 2 h, the blots were washed in TBST and developed using an enhanced chemiluminescence (ECL) detection kit (Bio-Rad) and visualized using a ChemiDoc Touch Imager (Bio-Rad). The bands on the blots were quantified using the ImageLab Software (Bio-Rad) and normalized for tubulin values as a loading control for densitometry analysis.

### Mitotracker red staining

HGF were seeded in an 8-well imaging chamber with coverslip bottom. After treatments as indicated in figure legends, cells were stained with MitoTracker™ Red CMXRos, a red-fluorescent dye that localizes in actively respiring mitochondria in live cells and its accumulation is dependent upon membrane potential. Briefly, cells were submitted to IL-1β stimulation with or without the pre-treatment with adenosine or EHNA + adenosine. Then, cells were stained with 200nM Mitotracker red dye in OptiMEM 30 mins at 37°C, protected from light. After washing with PBS 1x cells were fixed with 4% formaldehyde fixative solution for 15 min at room temperature and mounted in a mounting media containing DAPI to stain the nucleus. Images were obtained in 63x immersion oil objective lens in a Leica Stellaris Confocal microscope (Leica). Quantitation of MitoTracker Red was derived from confocal images using Fiji Software.

### Mitochondrial DNA copy number quantification

Total mitochondrial and nuclear genomic DNA was collected using PureLink Genomic DNA Mini Kit (Thermo Fisher Scientific), according to the manufacturer’s instructions. The purified total genomic DNA was quantified by quantitative real-time PCR, and the mtDNA level was normalized to that of nuclear DNA using the Human Mitochondrial DNA Copy Kit (Detroit R&D, Inc), according to the manufacturer’s instructions.

### ATP quantification

The amount of intracellular ATP was quantified using CellTiter-Glo 2.0 Assay (Promega) according to the manufacturer’s instructions.

### Statistical analysis

Statistical analysis was conducted for three independent experiments using the GraphPad Prism v9 software (GraphPad, San Diego, CA, USA) using ANOVA followed by multiple comparison tests. Data are presented as mean ± S.D. The cell number per well was chosen based on cell density optimization experiments for the specific assay. The significance level of p is indicated in each graph and in figure legends (*p <0.05; **p <0.01; *** p <0.001; ****p<0.0001).

## Results

### Extracellular adenosine dampens IL-1β-induced CXCL8 with a timely activation of A2AR/AMPK and in a mitochondrial-dependent fashion

**Figure 1:**
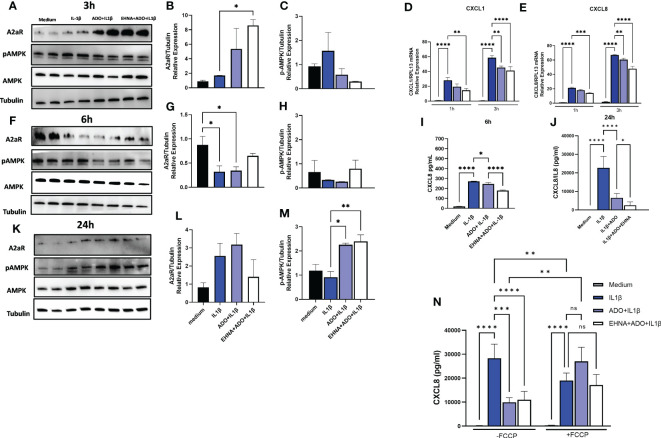
Adenosine dampens IL-1β-induced CXCL8 in a time-course activation of A2AR/AMPK and in a mitochondrial-dependent manner. Protein expression with the respective densitometry analysis of A2AR, pAMPK, AMPK and Tubulin in HGF stimulated with 1ng/mL IL-1β with or without 100µM adenosine (ADO) or 10µM EHNA+100µM ADO for **(A, B, C)** 3h; **(F, G, H)** 6h; or **(K, L, M)** 24h. **(D)** CXCL1 and **(E)** CXCL8 mRNA expression relative to RPL13A as a reference gene by RT-qPCR after 1h or 3h. **(I)** CXCL8 protein levels after 6h or **(J)** 24h of HGF stimulated with 1ng/mL IL-1β with or without 100µM adenosine (ADO) or 10µM EHNA+100µM ADO. **(N)** CXCL8 protein levels were measured in HGF supernatants after 24h of cells stimulated with 1ng/mL IL-1β with or without 100µM adenosine (ADO) or 10µM EHNA+100µM ADO in the presence or absence of pre-treatment with 1 µM FCCP. Data are presented as mean ± S.D. (*p <0.05; **p <0.01; ***p <0.001; ****p<0.0001). ns, not significant.

In a previous study, we have shown that IL-1β-induced CXCL8 secretion in gingival fibroblasts was dampened by the hydrolysis of extracellular ATP to adenosine through the activation of adenosine receptors (17). Furthermore, we demonstrated the involvement of AMPK activation in the adenosine anti-inflammatory effects in HGF (17). Here, we dissected the time-course dynamics of AMPK activation in the presence of adenosine (**Figure 1**). Our results show a time-dependent activation of AMPK when HGF were exposed to adenosine prior to IL-1β activation, resulting in decreased mRNA expression of CXCL1 and CXCL8 in early time points of 1h and 3h (**Figures 1D, E**) corroborating a decreased CXCL8 protein secretion in 6h and 24h (**Figures 1I, J**). We show that this occurs with the adenosine receptor A2A highest expression at the early time point of 3h, and with the maximum p-AMPK activation after 24h of adenosine exposure (**Figures 1A, F, K**). We also confirmed these results by pre-treating the cells with erythro-9-(2-Hydroxy-3-nonyl) adenine hydrochloride (EHNA), adenosine deaminase inhibitor, to prevent adenosine degradation in the media and allow it for more stability and sustained effects. When we shut down mitochondrion function by treating the cells with trifluoromethoxy carbonylcyanide phenylhydrazone (FCCP), a potent mitochondria uncoupler we could see adenosine lost its ability to downregulate IL-1β-induced CXCL8 (**Figure 1N**). These results demonstrate adenosine anti-inflammatory effects on gingival fibroblasts involves A2AR/AMPK axis and depends on mitochondrion function, which strongly supports the interplay between stromal cell mitochondrial metabolism and purinergic adenosine modulation of inflammation.

### Adenosine leads IL-1β-stimulated HGF to a metabolic switch towards an upregulated mitochondrial oxidative phosphorylation

**Figure 2:**
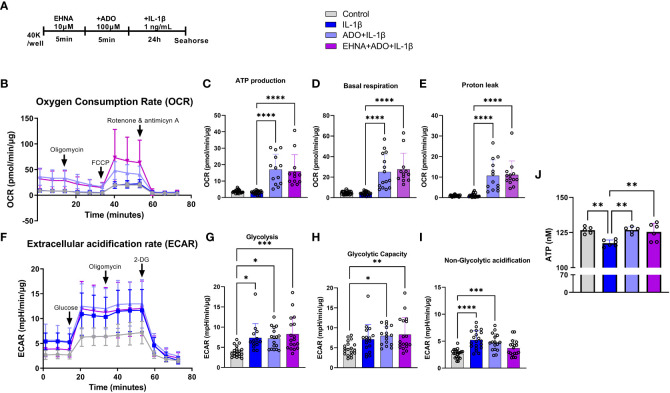
Adenosine leads IL-1β-stimulated HGF to a metabolic switch towards an upregulated mitochondrial oxidative phosphorylation. **(A)** HGF *in vitro* experimental model. **(B–E)** Oxygen Consumption Rate (OCR) of HGF during Seahorse Mito Stress Test in HGF stimulated with 1ng/mL IL-1β with or without 100µM adenosine (ADO) or 10µM EHNA+100µM ADO for 24h. **(F–I)** Extracellular acidification rate (ECAR) of HGF during Seahorse Glyco Stress test in HGF stimulated with 1ng/mL IL-1β with or without 100µM adenosine (ADO) or 10µM EHNA+100µM ADO for 24h. **(J)** ATP concentration (nM) measured by luminescence. Data are presented as mean ± S.D. (*p <0.05; **p <0.01; ***p <0.001; ****p<0.0001).

To facilitate a better understanding of how metabolic regulation interferes with cellular responses and the interplay between mitochondrial bioenergetics with inflammatory and immune reactions, we examined how adenosine regulates mitochondrial function in real time using seahorse analysis. Alterations in stromal cell metabolism are necessary to support the need for cellular homeostasis during inflammatory processes, cell injury or stressful cellular environment. In this context, metabolic reprogramming is thought to play a role as a critical event toward the transition of fibroblasts from quiescent to activated and aggressive cells, as in rheumatoid arthritis and cancer (33). Here, for the first time we observed a clear different metabolic profile of fibroblasts in the presence of adenosine or EHNA+adenosine when we submitted the cells to the mitochondrial stress test through a real-time seahorse analysis (**Figure 2**). A more in-depth analysis of the mitochondrial oxidative phosphorylation in gingival fibroblasts identified a higher oxygen consumption rate (OCR) (**Figure 2B**) when adenosine or EHNA+adenosine was added to the medium prior to IL-1β stimulation, reflected in a higher ATP production (**Figure 2C**), basal respiration (**Figure 2D**) and Proton leak (**Figure 2E**). We also evaluated the glycolytic metabolism of HGF in the presence of extracellular adenosine or EHNA+adenosine through the Glycolysis stress test (Agilent technology). Our data show adenosine and EHNA+adenosine do not affect the IL-1β-induced glycolysis (**Figure 2G**), glycolytic capacity (**Figure 2H**) or the non-glycolytic acidification (**Figure 2I**) which are all indicative of measurements of the Extracellular Acidification Rate (ECAR) (**Figure 2F**) higher in IL-1β-stimulated HGF. Additionally, we checked whether adenosine would change the rate of oxidation of each mitochondrial fuel by measuring mitochondrial respiration [or the OCR] of cells in the presence of fuel pathway inhibitors. Therefore, we measured the dependency, capacity and flexibility of cells to oxidize three mitochondrial fuels: Glucose (pyruvate), Glutamine (glutamate) and long-chain fatty acids (**Supplementary Figure 1**). Our data show no differences in dependency or capacity of HGF to oxidize any of these fuels in the presence or absence of adenosine (**Supplementary Figure 1**). Therefore, our results are indicative of an upregulated mitochondrial oxidative phosphorylation in HGF in the presence of adenosine, regardless of the mitochondrial fuel to maintain the higher OCR. We have also confirmed higher ATP concentration in adenosine-stimulated cells when compared with IL-1β only by quantifying intracellular ATP concentration by luminescence (**Figure 2J**).

### Adenosine increases mitochondrial biogenesis markers SIRT-1, PGC1α and upregulates PFKFB3 with no effect in other glycolysis-related markers

**Figure 3:**
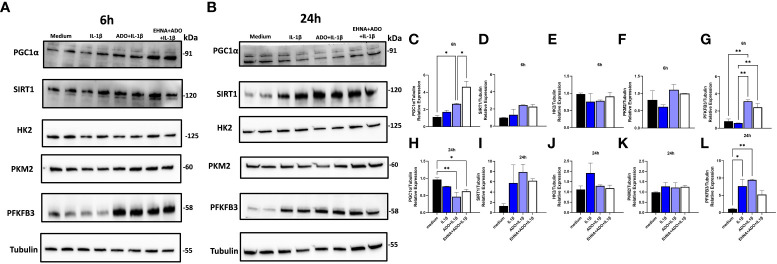
Adenosine increases mitochondrial biogenesis markers SIRT-1, PGC1α and upregulates PFKFB3 with no effect in other glycolysis-related markers. Protein expression with the respective densitometry analysis of oxidative phosphorylation or glycolysis markers after **(A, C–G)** 6h or **(B, H–L)** 24h of HGF stimulated with 1ng/mL IL-1β with or without 100µM adenosine (ADO) or 10µM EHNA+100µM ADO. Data are presented as mean ± S.D. (*p <0.05; **p <0.01).

Our next step was to estimate whether markers associated with increased mitochondrial function and markers related to glycolytic pathway would be altered after adenosine exposure. The peroxisome proliferator‐activated receptor‐γ coactivator (PGC)‐1α, has been extensively described as a master regulator of mitochondrial biogenesis (34). However, PGC-1α activity can be finely tuned in response to different metabolic situations. From this point of view, PGC-1α could be described as a mediator of the transcriptional outputs triggered by metabolic sensors, providing the idea that these sensors, together with PGC-1α, might be weaving a network controlling cellular energy expenditure (26). Two metabolic sensors, AMPK and SIRT1 have been described to directly affect PGC-1α activity and because we saw an upregulation of pAMPK earlier, we then checked the levels of PGC-1α and SIRT1, to confirm whether adenosine would be promoting mitochondrial biogenesis and thus maintaining mitochondrial function (35). We confirmed our hypothesis as we observed a higher PGC-1α expression in early 6h (**Figure 3A**) accompanied by a slightly higher SIRT1 in 24h (**Figure 3B**). We also saw a significantly higher expression of PFKFB3 in the presence of adenosine or EHNA+adenosine with 6h, but no change in other glycolytic molecules such as HK2 or PKM2 when compared to non-stimulated cells. Densitometry analysis of all immunoblots is shown in **Figures 3C–L**. These results confirmed adenosine stimulated mitochondrial biogenesis and fueled the need for more experiments to validate this hypothesis.

### Extracellular adenosine increases mitochondrial mass with preserved membrane potential

**Figure 4:**
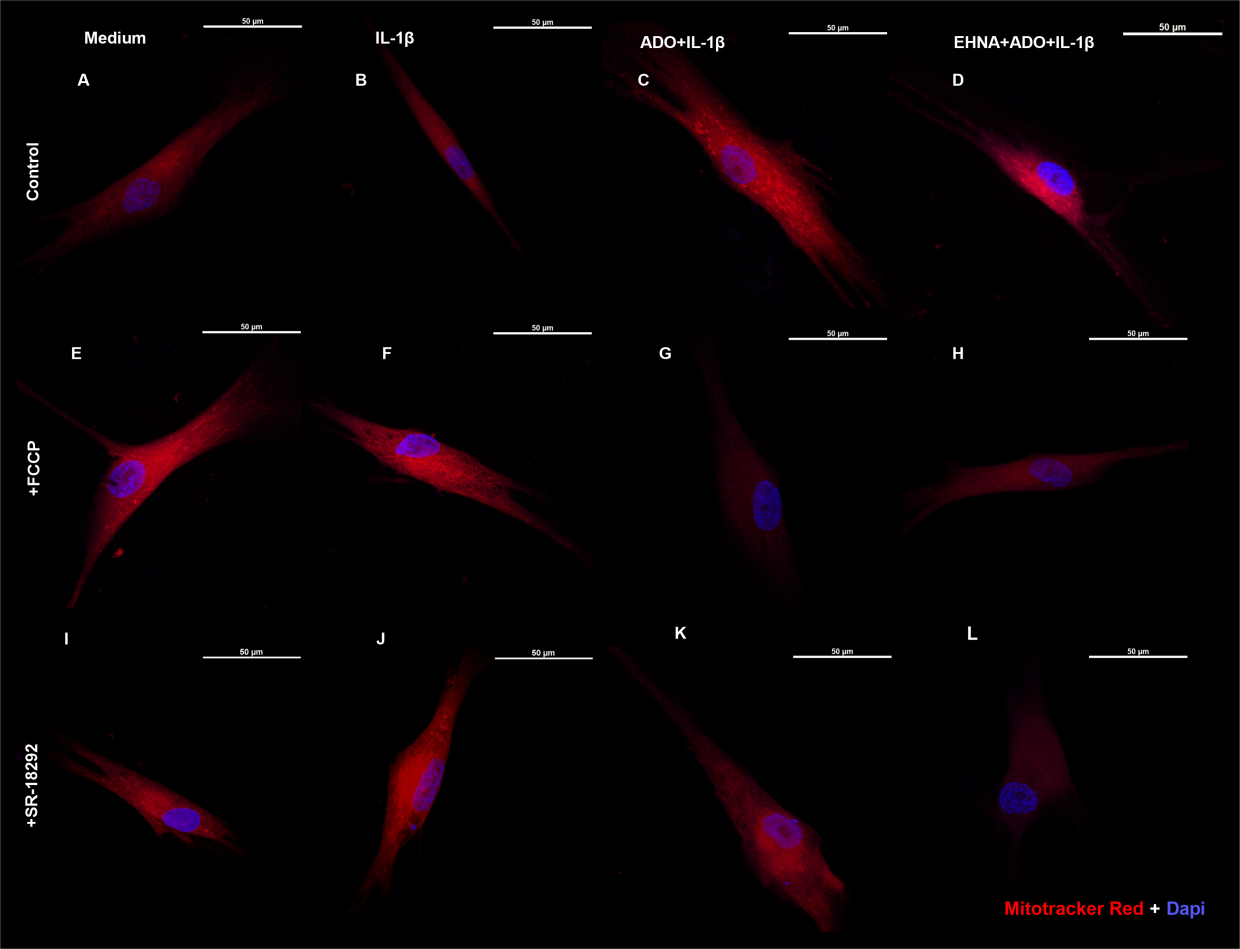
Extracellular adenosine increases mitochondrial mass with preserved membrane potential. Fluorescence microscopy of Mitotracker Red and DAPI staining of HGF stimulated with 1ng/mL IL-1β with or without 100µM adenosine (ADO) or 10µM EHNA+100µM ADO. **(A–D)** control non-treated cells. **(E–H)** pre-treated with 1µM FCCP 1h prior to subsequent stimulation. **(I–L)** pre-treated with 10µM SR-18292 1h prior to subsequent stimulation. Confocal images obtained at 63x immersion oil objective lens. Scale bar: 50µm.

We then investigated the mitochondrion mass with preserved membrane potential by staining HGF with Mitotracker Red (**Figure 4**) in the presence of FCCP, a well-known mitochondrial oxidative phosphorylation uncoupler (**Figures 4E–H**). In parallel, we also treated cells with SR-18292, a PGC-1 α inhibitor (**Figures 4I–L**). We analyzed mitochondrial staining in cells stimulated with IL-1β and pre-treated or not with adenosine or EHNA+adenosine under coupled (control) and uncoupled respiration conditions, wherein FCCP mitochondrial uncoupler was used to induce maximal respiration. FCCP treatment promotes mitochondrial depolarization, subsequently resulting in the fragmentation of mitochondrial networks (36), although mitochondrial fragmentation may require much higher concentrations. We confirmed adenosine and EHNA+adenosine boosted the mitochondrial staining in control non-stimulated cells, demonstrating a substantial increase in mitochondrial mass throughout the cell cytoplasm (**Figures 4C, D**). Our results showed less prominent mitotracker red staining of mitochondria from FCCP-treated cells, corroborating previously reported reduced mitochondrial mass (37). Furthermore, HGF treated with PGC-1 α inhibitor (SR-18292) dramatically decreased mitochondrial mass or demonstrated to have fewer actively respiring mitochondria, confirming the PGC-1α-dependent mitochondrial biogenesis elicited by adenosine. Quantification of the Mitotracker fluorescence intensity is provided in **Supplementary Figure 2**.

### Extracellular adenosine increases HGF mitochondrial biogenesis and decreases PINK-1 mediated mitophagy

**Figure 5:**
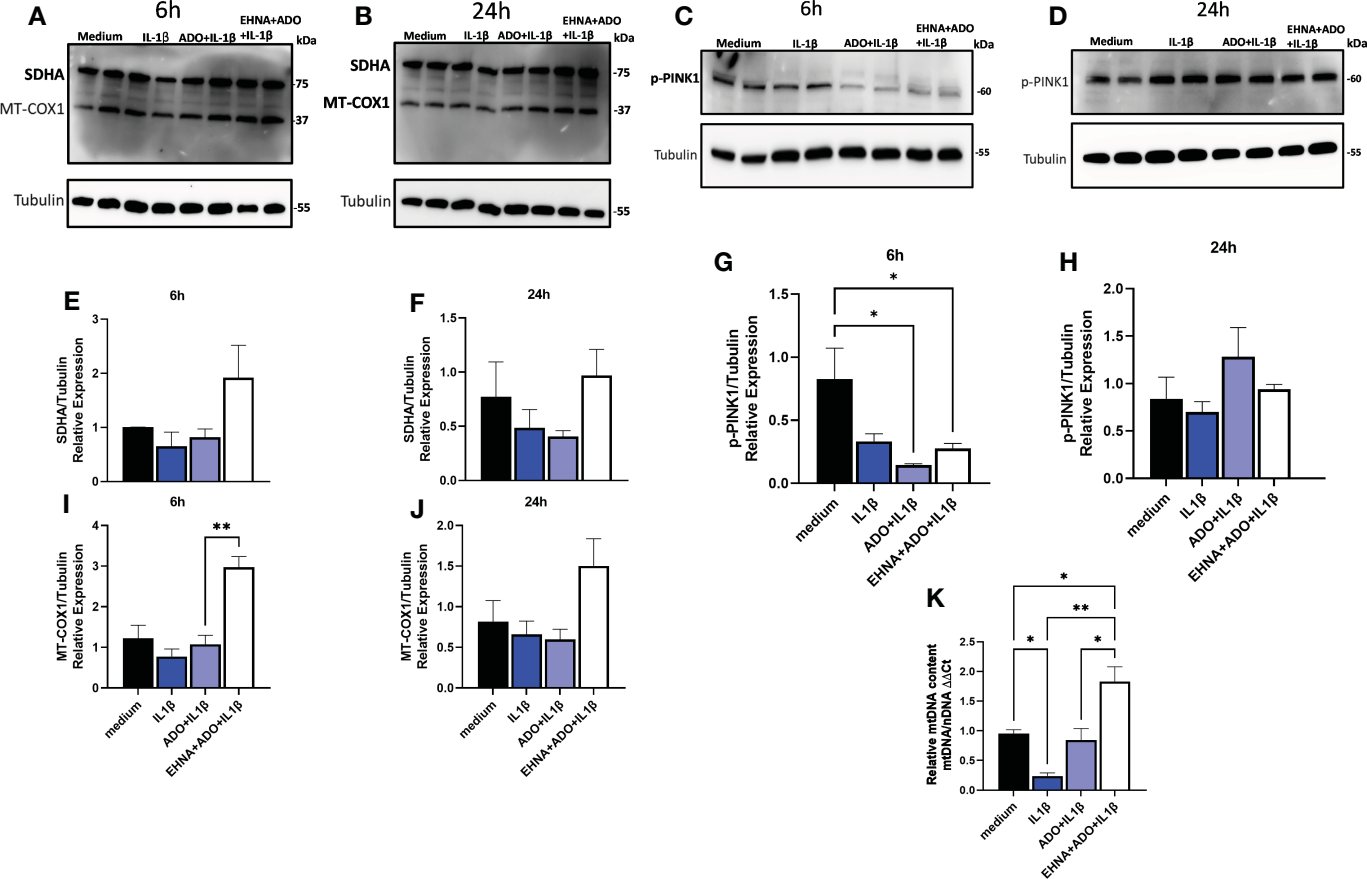
Extracellular adenosine increases HGF mitochondrial biogenesis and decreases PINK-1 mediated mitophagy. Protein expression with the respective densitometry analysis of mitochondrial biogenesis markers SDHA and MT-COX1 after **(A, E, I)** 6h or **(B, F, J)** 24h of HGF stimulated with 1ng/mL IL-1β with or without 100µM adenosine (ADO) or 10µM EHNA+100µM ADO. Protein expression with the respective densitometry analysis of p-PINK1 mitophagy marker after **(C, G)** 6h or **(D, H)** 24h. **(K)** Relative quantification of mtDNA/nDNA content by quantitative real time PCR. Data are presented as mean ± S.D. (*p <0.05; **p <0.01).

To further confirm the effect of adenosine exposure on the mitochondrial dynamics and to understand whether the adenosine-dependent effects in HGF were indeed affecting mitochondrial biogenesis, a MitoBiogenesis™ western blotting cocktail (Abcam) was used. The two main components of the cocktail target two proteins, which are each subunits of a different oxidative phosphorylation enzyme complex, one 37kDa subunit I of Complex IV (COX-I), which is mtDNA-encoded, and the 70kDa subunit of Complex II (SDH-A), which is nDNA-encoded (**Figures 5A, B**). Complex IV includes several proteins, which are encoded in the mitochondrion, while the proteins of Complex II are entirely encoded in the nucleus. After both 6h (**Figures 5A, E, I**) or 24h (**Figures 5B, F, J**) of exposure to our experimental conditions, we detected an increase of SDHA and MT-COX1, which was significant with EHNA+adenosine in the expression of mtDNA COX-1 subunit after 6h (**Figures 5A, I**). On the flip side, we also checked the time-course expression of PTEN-induced kinase 1 (p-PINK1), a molecular receptor of mitochondrial damage, which is particularly sensitive to depolarization of mitochondrial membrane potential and can recruit and phosphorylate ubiquitin-protein ligases to induce mitophagy (38). It has been previously reported p-PINK1 accumulates on dysfunctional mitochondria and its kinase activity is required for mitophagy (39). HGF stimulated fibroblasts treated with adenosine or EHNA+adenosine showed a clear decrease in p-PINK1 with 6h (**Figures 5C, G**) that was stabilized and not significant after 24h of exposure (**Figures 5D, H**). Additionally, we have quantified the mitochondrial DNA relative to the nuclear DNA content by quantitative real time PCR and our results confirmed increased DNA copy number in adenosine-stimulated cells compared to IL-1β only (**Figure 5K**). These results demonstrate adenosine not only stimulates stromal mitochondrial biogenesis but also dampens the dynamics of mitophagy, reprogramming cell bioenergetics towards mitochondrial function.

## Discussion

In this study we demonstrated for the first time that the anti-inflammatory effects of adenosine on IL-1β-induced CXCL8, reflecting modulation of hyper-inflammatory state of HGF, are driven by metabolic reprogramming. The role of the mitochondrial metabolism is this particular setting was through an increased mitochondrial mass and mtDNA, increased mitochondrial biogenesis and decreased expression of mitophagy-related marker in adenosine-exposed cells, ultimately affecting the underlying stromal inflammatory response. Furthermore, we demonstrated these effects involved upregulation of the AMPK/SIRT-1/PGC-1α-pathway.

Purinergic signaling has emerged as a key metabolic pathway that regulates immunity and inflammation (40) during chronic inflammatory conditions. The relevance of adenosine in the immune system has been established based on mounting scientific evidence showing that the nucleoside represents a paracrine inhibitor of inflammation, regulating the onset, extension, and termination of the inflammatory process and acting through adenosine receptors (41). Under conditions of cellular stress, inflammatory cells often shift to glycolytic production of ATP, which decreases the ATP-AMP ratio to activate AMPK and thus activates genes upregulating mitochondrial metabolism and oxidative phosphorylation, leading to reduced oxidative stress and inflammation (42). Studies have shown that the activation of AMPK is linked to anti-inflammatory effects, such as in mouse and human obesity models (42), and reduced oxidative stress in periodontitis models (43).

Macrophages have been classified into M1 phenotype (pro-inflammatory) or M2 phenotype (immunosuppressive) under different physiological conditions. LPS for example polarizes macrophages into M1, which are expected to produce high levels of pro-inflammatory mediators such as IL-1β. In contrast, M2 macrophages express high levels of IL-10 (44). These shifts are explained by metabolic shifts between oxidative phosphorylation and glycolysis (45). Our initial hypothesis was that the metabolic state of the ATP production, *via* preference of oxidative phosphorylation in the presence of adenosine, would be an important connection to the fibroblast capacity to influence periodontal homeostasis. This would involve mediation of a gradient of inflammatory chemokines such as CXCL8 under the influence of adenosine/AMPK signaling. So, it was interesting to see that in the presence of uncoupler agent FCCP, which increases respiration rate adenosine-anti-inflammatory effect on dampening CXCL8 was cancelled. This effect was accompanied by an aspect of mitochondrial reduced mass when we stained the cells with Mitotracker red. A recent report suggested treatment with FCCP resulted in mitochondrial fragmentation and reduced mitochondrial mass in healthy and diseased cell lines (37). In this study, although we have not shown mitochondrial fragmentation/fission, FCCP was used as a tool to test mitochondrial function and to mimic a physiological “energy demand” by stimulating the respiratory chain to operate at maximum capacity, which causes rapid oxidation of substrates (sugars, fats, and amino acids) to meet this metabolic challenge (46).

We hypothesized that similar to what was described for macrophages, the metabolic shift from glycolysis to oxidative phosphorylation of fibroblasts could directly influence their phenotype (from inflammatory to anti-inflammatory) in the presence of adenosine, and that this involved mitochondrial function. Nonetheless, our results show the adenosine-induced upregulation in mitochondrial oxidative phosphorylation (demonstrated by higher OCR values in our mito stress test) was not necessarily accompanied by decreased glycolysis in these cells according to our seahorse bioenergetic readouts. Our results show no change of the IL-1β-increased glycolysis in the presence of adenosine. In fact, our immunoblotting data confirms no change in markers of the glycolytic pathway such as HK2 and PKM2. The only upregulated marker was PFKFB3, which besides being a glycolytic activator has been linked to AMPK activation, mechanisms of regeneration and repair following injury and promotion of oxidative phosphorylation (47), which we believe might be the case here. We believe PFKFB3 might have additional roles not related to the regulation of glycolysis and we show here it can be directly influenced by purinergic adenosine signaling. These results prompted our attention to further investigate whether adenosine could possibly be inducing a differential modulation of the mitochondrial fuel, such as dependency or preference of oxidation for one of the three mitochondrial fuels (glucose, glutamine or fatty acid), which was revealed not to be the case as demonstrated here.

Our data show the increase of the mitochondrial biogenesis marker PGC-1α and decrease in mitophagy marker p-PINK1, which was the main piece of evidence that adenosine was directly modulating mitochondrial dynamics by favoring biogenesis over mitophagy and therefore sustaining its anti-inflammatory effects. It is important to highlight there are other pro-fission proteins regulating mitophagy machinery such as Dynamin-related protein 1 (DRP-1), for example. Thus, future studies are needed to further validate the mechanisms of adenosine in regulating pro-fission or anti-fission activity which can be highly dependent on differences in DRP-1 phosphorylation (48).

An upregulation in PCG-1α *via* AMPK activation leads to increase in mitochondrial oxidative respiration, and this gradual overexpression of PCG-1α leads to decreased NF-κB activity (49). In models of skeletal muscle during exercise, SIRT1 is activated and upregulates PCG-1α activity through deacetylation to stimulate genes for fat oxidation; however, this energy depletion through exercise also activates AMPK, which further activates PCG-1α. In a reciprocal positive regulation mechanism, AMPK upregulates SIRT1 activity and SIRT1 activates AMPK by deacetylating liver kinase B1 (LKB1) (50).

Though complex, the relationship between these proteins is important in the regulation of mitochondrial biogenesis and inhibition of inflammatory pathways. SIRT1 and AMPK can inhibit NF-κB activity separately and through mutual activation. SIRT1 deacetylates Lys310 on the p65 subunit of NF-κB to inhibit it, while AMPK can inhibit NF-κB activity through activation of SIRT1 and Forkhead box O 3a (FoxO3a) transcription factor that led to the expression of kB-Ras1 which also inhibits NF-κB (49). Therefore, through all these possible mechanisms we could link the impaired NF-κB activation to the decreased CXCL8 levels elicited by adenosine. While AMPK has been implicated in the activation of PCG1-α *via* SIRT1 upregulation (50), AMPK can also activate PCG-1α through the p38 mitogen-activated protein kinase (MAPK) and Histone deacetylase (HDAC) pathways (25), indicating a complex relationship between these proteins.

An important implication of these findings would be to see the impact of adenosine-associated modulation of stromal mitochondrial biogenesis in aging-related sustained inflammatory diseases such as periodontitis. PGC-1α expression is reduced with aging (51) and mitochondrial dysfunction has become a key hallmark among the factors that contribute to aging, being associated with the development of numerous age-related diseases (52). In addition, fibroblasts are essential stromal cells for gingival architecture and function and in other tissues, such as skin these cells lose their functional specialization and change their phenotype with aging (53).

**Figure 6:**
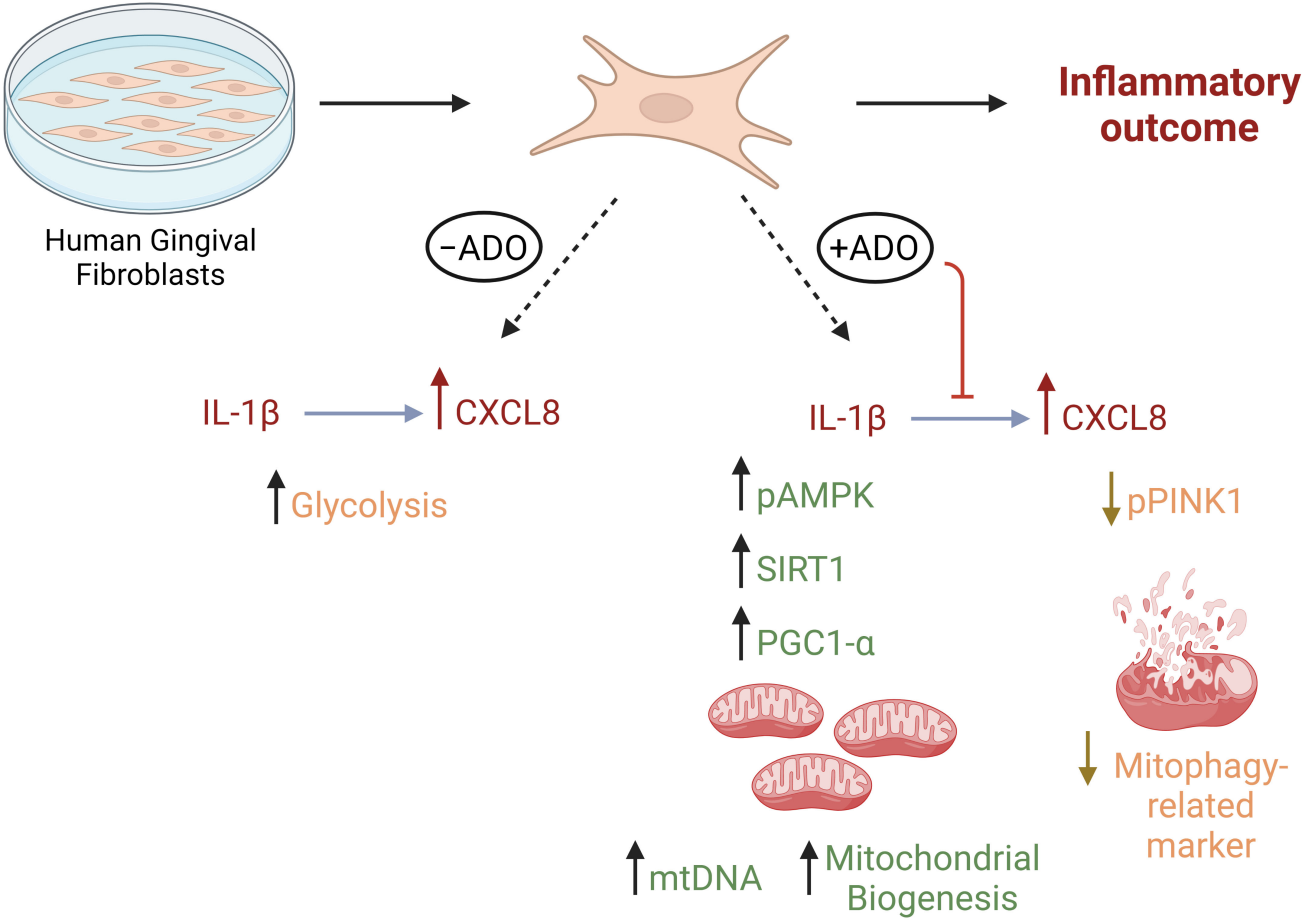
Metabolic reprogramming through mitochondrial biogenesis drives adenosine anti-inflammatory effects in gingival stromal cells. This figure summarizes our findings regarding metabolic effects of Il-1β-activated fibroblast response in the presence or absence of adenosine. Created in Biorender.com.

We (1, 54) and others (55) have shown that fibroblasts respond differently to inflammatory stimuli such as bacterial lipopolysaccharide and TLR ligands depending upon their site of origin. In other models such as arthritis, Croft AP et al. (56) described functionally distinct fibroblast subsets that do not overlap in function in regards to modulation of tissue damage. Regarding human gingiva, recent study by Caetano AJ et al. (57) demonstrated a spatial localization of pro-inflammatory CXCL8-hyper secreting fibroblasts in human gingiva using single-cell technology. The findings of the present study reveal a new mechanism linking adenosine anti-inflammatory effects through mitochondrial activity with regards to CXCL8 chemokine which is highly relevant for periodontal pathogenesis. Whether the findings of this study will be extended to other gingival fibroblast states or subtypes remains unclear and our follow up studies will help to understand this. We summarize our findings in **Figure 6**.

Besides the intricacy of these pathways, this study brings novel perspective to the effects of purine metabolism in metabolic reprogramming of fibroblasts, namely the role of mitochondrial biogenesis in the underlying inflammatory phenotype of fibroblasts. It also enlightens the therapeutic possibility of targeting pathways of energy metabolism in gingival stromal cells, with particular emphasis on the intersection between inflammation, purinergic signaling and its relationship with mitochondrial metabolism.

## Conclusions

Our study reveals a novel perspective for understanding the interplay between stromal cell biology, purinergic signaling and mitochondrial bioenergetics in regulation of IL1β-mediated inflammation. Stromal hyper-inflammatory response typified by CXCL8 secretion is modulated by the adenosine/AMPK/SIRT-1/PGC-1α axis through mitochondrial biogenesis. Therapeutically targeting this pathway in gingival fibroblasts might be a promising future strategy to control stromal-mediated sustained hyper-inflammatory responses.

## Data availability statement

The original contributions presented in the study are included in the article/**Supplementary Material**. Further inquiries can be directed to the corresponding author.

## Author contributions

ACM contributed to conception, design of the study, mentored and supervised experiments. ER-J, YH and CWC contributed to data interpretation, intellectual input and revision. NP, SD, WR, RS-L performed the experiments, statistical analysis and prepared the figures. RM contributed with cells, protocol approvals and critically revised the manuscript. NP wrote sections of the manuscript. ACM wrote the first draft of the manuscript. All authors contributed to the article and approved the submitted version.

## Supplementary material

Supplementary Figure 1 Adenosine does not change mitochondrial fuel dependency or capacity to maintain the Oxygen Consumption Rate (OCR). HGF stimulated with or without 10µM EHNA + 100µM ADO for 24h were submitted to a fuel oxidation test through seahorse analysis. Measurement of fuel dependency was determined on the reliance of cells in a particular fuel (glucose, glutamine or fatty acid) to maintain baseline OCR. Capacity was measured based on the ability of cell mitochondria to oxidize a fuel when others are inhibited. Data are presented as mean ± S.D and represent the percentage of each fuel oxidation in the presence or absence of ADO.

Supplementary Figure 2 Quantification of Cell Fluorescence Intensity for the Mitotracker Red confocal images presented in **Figure 4**. Data are presented as mean ± S.D and represent the average of 3 independent experiments. (*p <0.05; **p <0.01).
