# Supplementary figures and images for: Metabolic reprogramming through mitochondrial biogenesis drives adenosine anti-inflammatory effects: new mechanism controlling gingival fibroblast hyper-inflammatory state
Source: Front Immunol. 2023 Jun 7;14:1148216. doi: 10.3389/fimmu.2023.1148216 (PMC10282177; doi:10.3389/fimmu.2023.1148216)

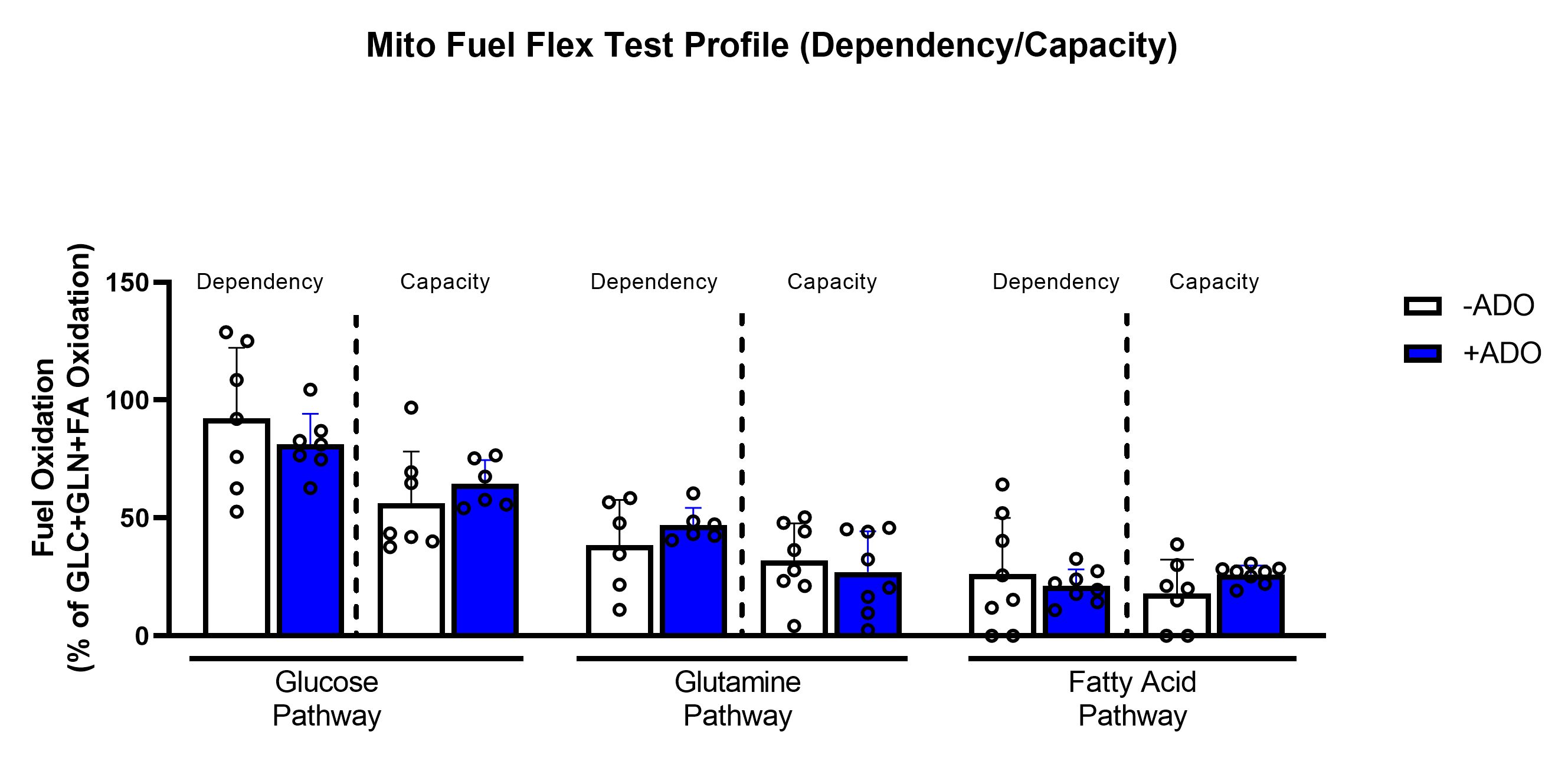

Supplement: Supplementary Figure 1 — Adenosine does not change mitochondrial fuel dependency or capacity to maintain the Oxygen Consumption Rate (OCR). HGF stimulated with or without 10µM EHNA + 100µM ADO for 24h were submitted to a fuel oxidation test through seahorse analysis. Measurement of fuel dependency was determined on the reliance of cells in a particular fuel (glucose, glutamine or fatty acid) to maintain baseline OCR. Capacity was measured based on the ability of cell mitochondria to oxidize a fuel when others are inhibited. Data are presented as mean ± S.D and represent the percentage of each fuel oxidation in the presence or absence of ADO. [file Image_1.tif]

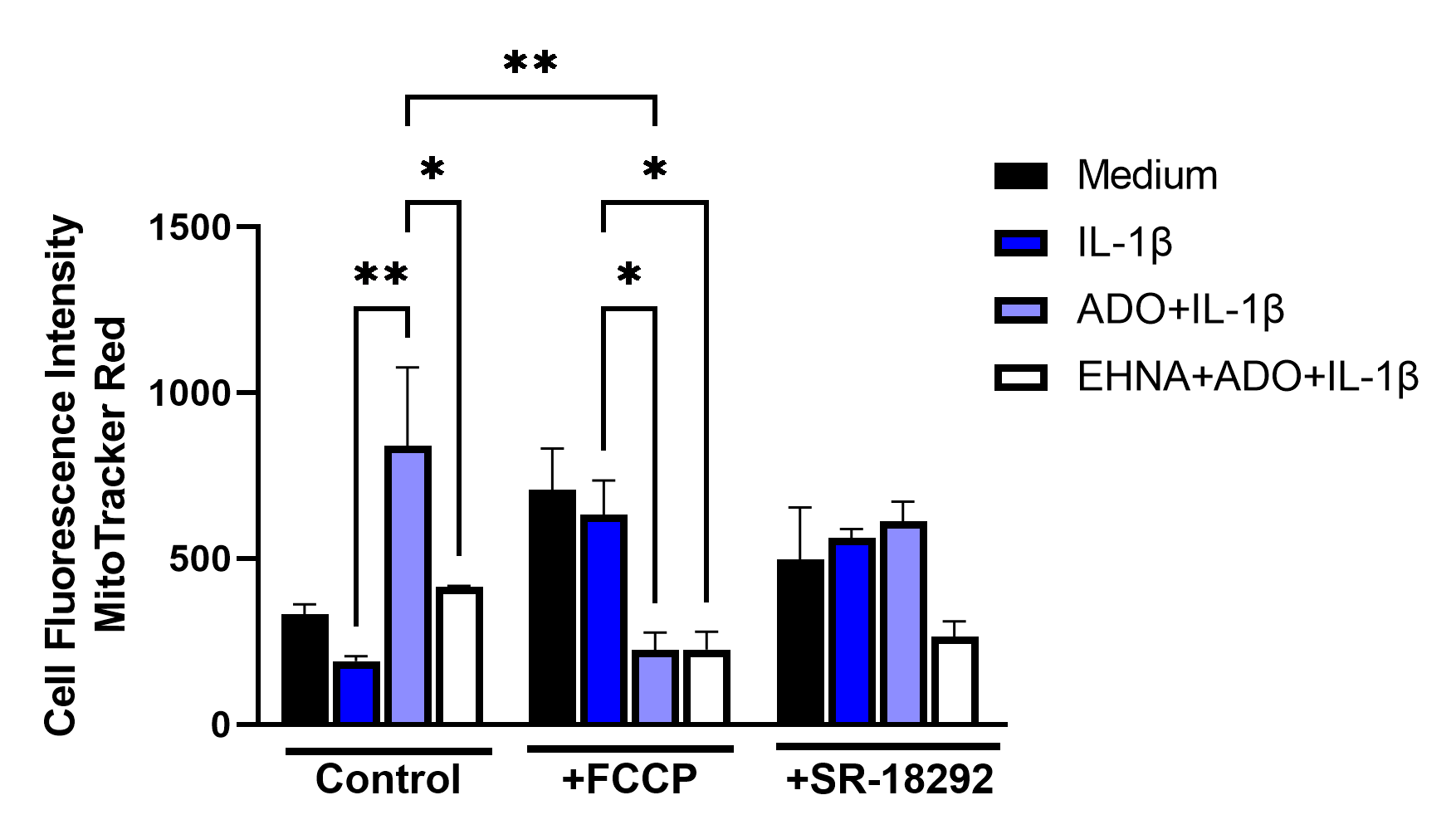

Supplement: Supplementary Figure 2 — Quantification of Cell Fluorescence Intensity for the Mitotracker Red confocal images presented in Figure 4 . Data are presented as mean ± S.D and represent the average of 3 independent experiments. (*p <0.05; **p <0.01). [file Image_2.tif]
